# Effect of dietary interventions on kidney function in adults with obesity without chronic kidney disease: a systematic review and meta-analysis of randomized controlled trials

**DOI:** 10.3389/fnut.2026.1836822

**Published:** 2026-06-29

**Authors:** Weilong Su, Haicheng Gou, Lingfeng Yuan, Yisheng Luan, Yaxin Wang, Xiaobo Song, Yingzhe Xiong

**Affiliations:** 1 School of Physical Education and Sports, Central China Normal University, Wuhan, China; 2 Division of Sports Science and Physical Education, Tsinghua University, Beijing, China; 3 Physical Education Institute, Putian University, Putian, China

**Keywords:** dietary interventions, glomerular filtration rate, kidney function, meta-analysis, obesity

## Abstract

**Objective:**

This study aimed to systematically evaluate the effects of dietary interventions on kidney function in adults with obesity without chronic kidney disease (CKD).

**Methods:**

A comprehensive literature search was conducted in PubMed, Web of Science, Embase, Cochrane Library for randomized controlled trials. Random-effects meta-analyses calculated standardized mean difference (SMD), with subgroup analyses by glomerular filtration rate (GFR) assessment method (estimated GFR [eGFR] vs. measured GFR [mGFR]).

**Results:**

Eighteen trials involving 1,438 participants were included. Dietary interventions showed no significant effect on GFR (SMD: 0.06; 95% CI: −0.20 to 0.32; *p* = 0.642). In subgroup analyses, eGFR increased significantly whereas mGFR showed a non-significant reduction (*p* = 0.0005 for subgroup differences). No significant effect was found for urinary albumin excretion rate (UAER) (SMD: −0.04; 95% CI: −0.39 to 0.30; *p* = 0.765). Creatinine clearance (CrCl) showed a significant increase (SMD: 0.22; 95% CI: 0.03 to 0.41; *p* = 0.030).

**Conclusion:**

Dietary interventions showed inconsistent effects across kidney function measures in adults with obesity without CKD. Although significant improvements were observed in certain kidney function-related indices (eGFR and CrCl), the available evidence did not indicate improved true filtration or reduced urinary albumin excretion. Importantly, these findings suggest that the apparent renal effects of dietary intervention depend on how kidney function is assessed.

**Systematic review registration:**

https://www.crd.york.ac.uk/PROSPERO/view/CRD420261297927, CRD420261297927.

## Graphical abstract

**Figure:**
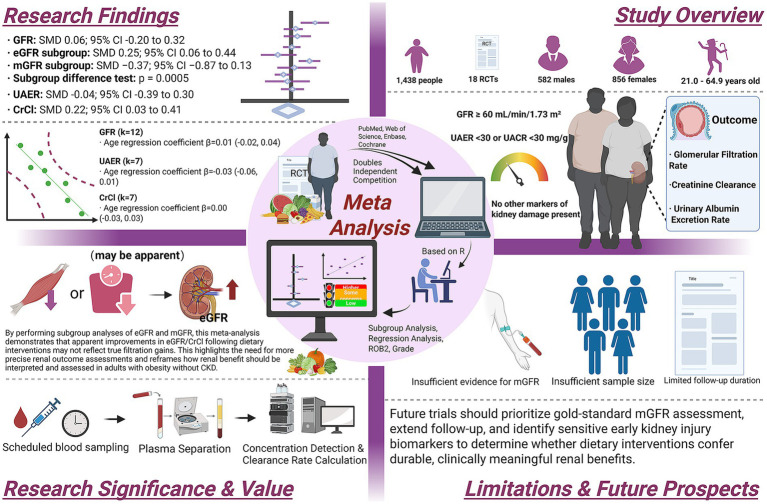
Created with BioRender.com.

## 1 Introduction

Chronic kidney disease (CKD) is a major and growing contributor to premature mortality worldwide, affecting approximately 788 million adults (1). Projections suggest that CKD will become the fifth leading cause of years of life lost worldwide by 2040 (2), highlighting the need to identify effective early preventive strategies targeting modifiable drivers of disease progression. Obesity is an established, independent risk factor for CKD (3). In a large meta-analysis including 4,965,285 participants, metabolically healthy obesity (RR: 1.47; 95% CI: 1.31 to 1.65) was associated with a higher risk of having CKD (4). This association may be driven by multiple obesity-related mechanisms, including glomerular hyperfiltration (GH), overactivation of the renin-angiotensin-aldosterone system (RAAS), insulin resistance and hyperinsulinemia, elevated levels of pro-inflammatory cytokines, as well as ectopic lipid deposition and lipotoxicity (5).

Kidney Disease: Improving Global Outcomes (KDIGO) emphasized that early-onset obesity and prolonged exposure to obesity carry the highest risk of developing CKD (6). Accordingly, adults with obesity but without CKD represent a large, high-risk population at a potentially modifiable stage, in which timely lifestyle intervention may interrupt the pathophysiological processes driving obesity-related glomerular hyperfiltration and reduce the likelihood of progressive kidney damage.

Early obesity-related kidney stress is largely mediated by hemodynamic and metabolic pathways that are potentially reversible through dietary modification. However, evidence regarding the effect of dietary interventions on kidney function and albuminuria in adults with obesity without CKD remains unclear. Although KDIGO provides the specific dietary recommendations for CKD patients, it does not specifically address adults with obesity without CKD (7). Most existing meta-analyses were conducted in populations with CKD. In contrast, only two earlier meta-analyses (published in 2014 and 2016) focused on adults with obesity without CKD (8, 9). However, both of them combined creatinine clearance (CrCl), estimated glomerular filtration rate (eGFR), and measured glomerular filtration rate (mGFR) into a single glomerular filtration rate (GFR) outcome and defined CKD solely by a GFR threshold of <60 mL/min/1.73 m^2^. This approach is methodologically problematic. First, CrCl is not interchangeable with GFR. Because creatinine is filtered by the glomerulus but is also secreted by the proximal tubule, CrCl systematically overestimates true GFR by approximately 10 to 40% at all levels of GFR (especially at higher GFR) (10). More importantly, dietary interventions could lead to weight loss, reduction in muscle mass, and decreased creatinine generation. As a result, renal indices that rely on creatinine, such as creatinine-based eGFR and CrCl, may show an apparent increase that does not reflect true filtration changes (11). In addition, KDIGO defined CKD as GFR < 60 mL/min/1.73 m^2^ or albuminuria (ACR ≥ 30 mg/g or AER ≥ 30 mg/24 h) or other markers of kidney damage present (7). Therefore, a GFR-only definition may miss earlier CKD with preserved GFR but albuminuria. Taken together, these limitations highlight the need to separate mGFR from creatinine-based estimates and to incorporate albuminuria to clarify the true renal effects of dietary interventions in obesity without CKD.

Accordingly, we conducted a comprehensive systematic review and meta-analysis of randomized controlled trials evaluating the effects of dietary interventions in adults with obesity without CKD. In this study, we analyzed renal outcomes separately according to assessment method (mGFR, eGFR, and CrCl) and also examined the urinary albumin excretion rate (UAER) to provide a robust assessment of early kidney damage. By separately analyzing mGFR, eGFR, CrCl, and UAER, this study aims not only to resolve previous methodological inconsistencies but also to provide insight into early obesity-related renal alterations and a more accurate assessment of the renal effects of dietary interventions in this high-risk population.

## 2 Methods

This systematic review was conducted following the Preferred Reporting Items for Systematic Reviews and Meta-analyses (PRISMA) statement (12) (Supplementary Table S1) and was registered with PROSPERO (CRD420261297927).

### 2.1 Search strategy

Two authors (WS and HG) independently searched PubMed, Web of Science, Embase, Cochrane Library from database inception to 13 January 2026. The detailed search strategy is provided in Supplementary Table S2. To ensure comprehensive coverage, we also conducted manual searches of the reference lists of included articles and relevant reviews.

### 2.2 Eligibility criteria

To be eligible for inclusion, studies had to be randomized controlled trials enrolling adults (aged ≥18 years) with obesity (BMI ≥ 30 kg/m^2^) that compared dietary interventions with conventional diet, standard care, or no intervention. To focus on individuals without Chronic kidney disease (CKD), we excluded trials involving participants with CKD, defined as GFR < 60 mL/min/1.73 m^2^ or albuminuria (AER ≥ 30 or ACR ≥ 30 mg/g [≥3 mg/mmol]) or other markers of kidney damage present (7). Eligible trials were required to report quantitative kidney function outcomes, including GFR (estimated or measured), UAER and/or CrCl. Trials were excluded if the effect of the dietary intervention could not be isolated from other concurrent interventions.

### 2.3 Study selection

Records retrieved from database searches were imported into EndNote and deduplicated. Two reviewers (WS and HG) independently screened the titles and abstracts of retrieved studies. Potentially relevant articles were retrieved for full-text review against the eligibility criteria. Any disagreements were resolved by discussion or consultation with a third reviewer (YX).

### 2.4 Data extraction

Study characteristics and relevant data were extracted independently and in duplicate by two reviewers (WS and HG) using a standardized extraction form. Extracted data included: first author, publication year, country, sample size, participant characteristics (sex, age, BMI, and baseline metabolic status), intervention details (type, duration, and macronutrient composition), and renal outcomes (GFR, UAER and CrCl). Discrepancies were resolved through discussion or consultation with a third reviewer (YX). For each included study, we extracted the means and standard deviations (SDs) of post-intervention outcomes. If the post-intervention values were unavailable, they were calculated using the following formulas, with the correlation coefficient set to 0.5 (13):

Meanpost=Meanpre+Meanchange,SDpost=2×Corr×SDpre+4×Corr2×SDpre2–4×(SDpre2−SDchange2)2

When numerical results were not reported, data were extracted from Figures using WebPlotDigitizer (14). In cases of missing or incomplete data, we contacted the corresponding authors for the information.

### 2.5 Risk of bias assessments

We used the revised Cochrane risk of bias (RoB 2) tool for randomized controlled trials to assess the risk of bias in the included studies (15). Two reviewers (WS and HG) independently performed the bias risk assessment across five domains: the randomization process, deviations from the intended interventions, missing outcome data, measurement of the outcome, and selection of the reported result. Any disagreements were resolved by discussion or consultation with a third reviewer (YX). Trials were judged as low risk of bias if all domains were assessed as low risk; as some concerns if at least one domain was assessed as some concerns but no domain was assessed as high risk; and as high risk of bias if at least one domain was assessed as high risk.

### 2.6 Certainty of evidence

We used the Grading of Recommendation Assessment, Development, and Evaluation (GRADE) approach to evaluate the overall certainty of evidence. Each included outcome (GFR, UAER, and CrCl) was rated as having high, moderate, low, or very low quality of evidence, based on five domains: risk of bias, inconsistency, indirectness, imprecision, and publication bias (16). Disagreements between the two reviewers (WS and HG) were addressed through discussion or consultation with a third reviewer (YX).

### 2.7 Statistical analyses

All analyses were conducted using two-sided tests with a significance level of *α* = 0.05 in R (version 4.5.1) with the meta, dmetar, and metafor packages. Continuous outcomes (GFR, UAER, and CrCl) were summarized as standardized mean differences (SMDs) calculated using Hedges’ g. In multi-arm randomized controlled trials, all eligible intervention arms from the same trial were assigned a common study identifier. We then followed Cochrane guidance to account for the correlation arising from the shared control group, thereby avoiding double-counting and over-inflation of study weights (13). Pooled effects were estimated using random-effects models with the DerSimonian-Laird estimator for between-study variance (τ^2^) (17), and are reported with 95% confidence intervals (CIs) and two-sided *p* values. The magnitude of SMDs was interpreted as minimal (|SMD| ≤ 0.2), small (0.2 < |SMD| ≤ 0.5), moderate (0.5 < |SMD| ≤ 0.8), and large (|SMD| > 0.8).

To explore potential sources of heterogeneity and assess robustness, we performed sensitivity analyses using influence diagnostics. Prespecified subgroup analyses examined whether the effects on GFR differed according to the method used to assess kidney function (eGFR vs. mGFR). In addition, random-effects meta-regression was conducted using restricted maximum likelihood (REML) with the Hartung–Knapp adjustment, with baseline age included as a covariate scaled per 10-year increase. Publication bias was assessed using contour enhanced funnel plots and Egger regression test, with *p* > 0.05 indicating no evidence of asymmetry (18).

## 3 Results

### 3.1 Study selection and characteristics

**Figure 1:**
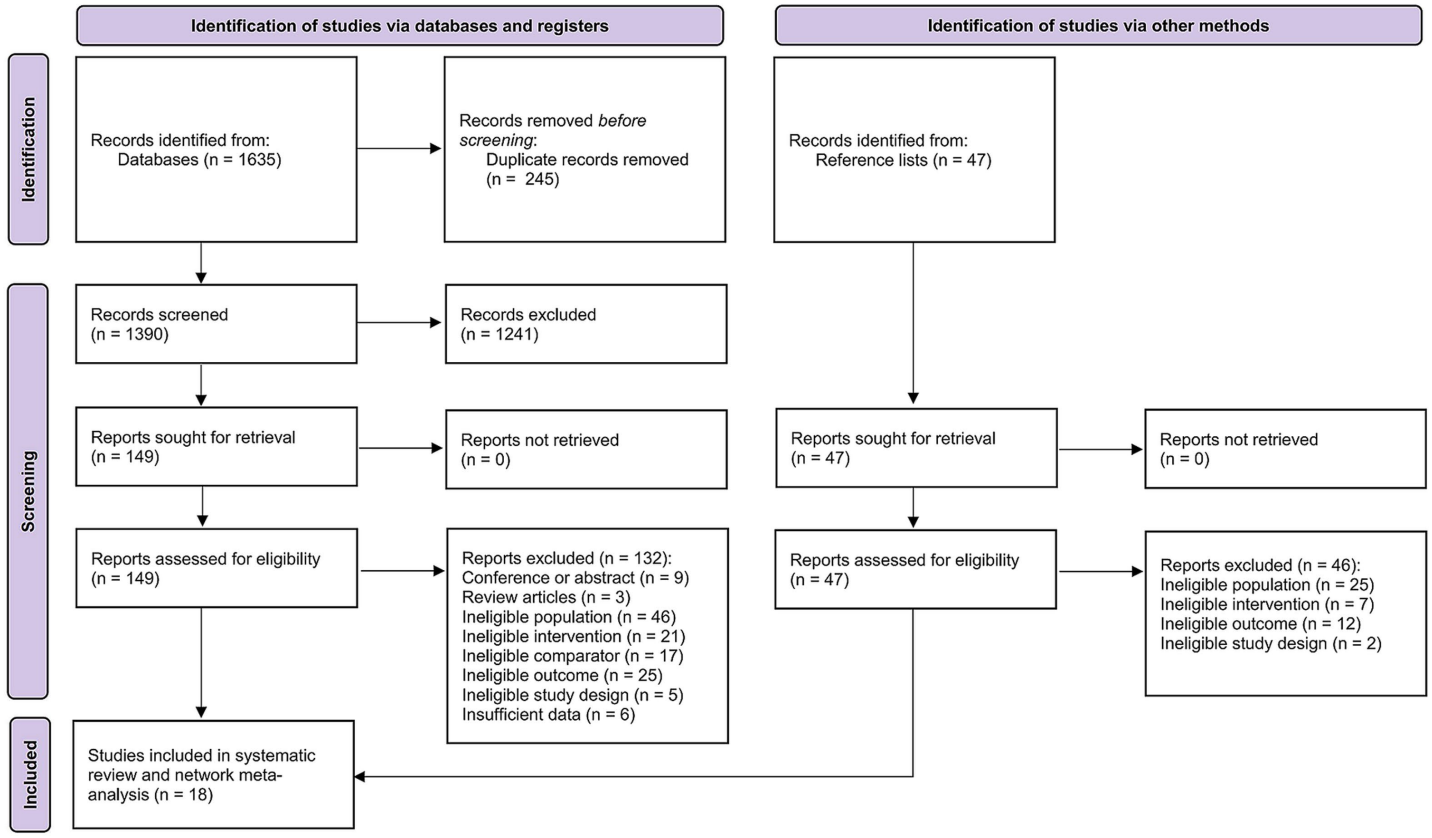
Flow diagram of the study selection process.

We identified 1,635 records from database searches. After removing 245 duplicates, 1,390 records were screened by title and abstract, resulting in the exclusion of 1,241 records. Following the full-text assessment of 149 articles and 47 additional records identified through reference list screening, 18 studies with a total of 1,438 participants were included (Figure 1).

All included studies were published between 1999 and 2025. Of the 18 studies, 9 were conducted in Europe, 6 in North America, and 3 in Australia. Among these 1,438 participants, 582 (40.5%) were male and 856 (59.5%) were female. The mean age across the included studies ranged from 21.0 to 64.9 years. The mean intervention duration was 35.4 weeks, ranging from 3 weeks to 2 years. Regarding baseline metabolic status, 50.6% of participants had uncomplicated obesity and 22.9% had type 2 diabetes, while the remainder exhibited mixed metabolic disorders or other comorbidities (Table 1).

**Table 1:** Characteristics of included studies.

Study	Country	Number (M/F)	Age	BMI	Interventions	Duration (weeks)	Baseline Metabolic Status	Outcome	Macronutrient Composition
Abbate et al. 2021 (31)	Spain	43 (27/16)	52.3 ± 7.1	34.3 ± 3.9	Mediterranean diet	26	NAFLD	eGFR	40–45% carbohydrate, 25% protein, and 30–35% fat
		42 (24/18)	54.1 ± 8.9	33.4 ± 3.7	Conventional diet				45–65% carbohydrate, 10–35%protein, and 20–35% fat
Brinkworth et al. 2010 (32)	Australia	33 (11/22)	51.5 ± 7.7	33.6 ± 4.0	Low carbohydrate diet	52	MetS risk factors	eGFR	4% carbohydrate, 35% protein, and 61% fat
		35 (14/21)	51.5 ± 7.7	33.5 ± 3.8	Conventional diet				46% carbohydrate, 24% protein, and 30% fat
Dutheil et al. 2012 (33)	France	14 (9/5)	60.6 ± 6.0	35.2 ± 4.2	High-protein diet	26	MetS	CrCl	Protein intake: 1.2 g/kg/day
		14 (10/4)	62.9 ± 6.9	32.1 ± 4.2	Normal-protein diet				Protein intake: 1.0 g/kg/day
Friedman et al. 2012 (34)	USA	153 (50/103)	46.2 ± 9.2	36.1 ± 3.6	Low carbohydrate diet	104	Uncomplicated obesity	CrCl	Carbohydrate intake: 20 g/day
		154 (49/105)	44.9 ± 10.2	36.1 ± 3.5	Conventional diet				55% carbohydrate, 15%protein, and 30% fat
Gannon et al. 2003 (35)	USA	12 (10/2)	61 ± 12.2	31 ± 4.6	High-protein diet	5	T2DM	UAER, CrCl	40% carbohydrate, 30% protein, and 30% fat
		12 (10/2)	61 ± 12.2	31 ± 4.6	Normal-protein diet				55% carbohydrate, 15% protein, and 30% fat
Gils Contreras et al. 2018 (36)	Spain	43 (14/29)	45.2 ± 10.5	47.3 ± 5.3	Calorie restriction	3	Obesity-related comorbidities	eGFR	46.8% carbohydrates, 36.4% protein, 9.3% fat, and 7.4% fiber
		41 (7/34)	45.5 ± 9.7	47.2 ± 5.0	Conventional diet				46.8% carbohydrates, 36.4% protein, 9.3% fat, and 7.4% fiber
Goday et al. 2016 (37)	Spain	45 (15/30)	54.8 ± 8.8	33.3 ± 1.5	ketogenic diet	16	T2DM	eGFR	Carbohydrate intake: < 50 g/day
		44 (16/28)	54.2 ± 8.0	32.9 ± 1.6	Conventional diet				45–60% carbohydrate, 10–20% protein, and < 30% fat
Lambert et al. 2017 (38)	Australia	6 (6/0)	24 ± 3.2	31.9 ± 6	Calorie restriction	26	Uncomplicated obesity	CrCl	48% carbohydrate, 22% protein, and 30% fat
		10 (10/0)	21 ± 2.4	29.2 ± 2.2	Conventional diet				NR
Leidy et al. 2007 (39)	USA	25 (0/25)	46 ± 9.2	30.7 ± 4.1	High-protein diet	12	Uncomplicated obesity	eGFR	45% carbohydrate, 30% protein, and 25% fat
		21 (0/21)	53 ± 15	30.5 ± 3	Normal-protein diet				57% carbohydrate, 18% protein, and 25% fat
Li et al. 2010 (40)	USA	44 (8/36)	48.9 ± 11.8	34.7 ± 6.8	High-protein diet	52	Uncomplicated obesity	CrCl	40% carbohydrate, 30% protein, and 30% fat
		41 (15/26)	49.7 ± 9.1	34.3 ± 10.3	Normal-protein diet				55% carbohydrate, 15% protein, and 30% fat
Luger et al. 2013 (41)	Austria	22 (8/14)	61.0 ± 5.7	33.0 ± 4.2	High-protein diet	12	T2DM	eGFR, UAER	40% carbohydrate, 30% protein, and 30% fat
		22 (6/16)	63.7 ± 5.2	33.6 ± 5.3	Normal-protein diet				55% carbohydrate, 15% protein, and 30% fat
Nachon Garcia et al. 2025 (42)	Mexico	51 (7/44)	36.6 ± 9.3	32.7 ± 2.0	ketogenic diet	12	Uncomplicated obesity	eGFR	Carbohydrate intake: < 60 g/day
		26 (4/22)	37.7 ± 11.2	32.4 ± 1.6	Conventional diet				50% carbohydrate, 25% protein, and 25% fat
Nuttall & Gannon, 2006 (43)	USA	8 (0/8)	63 ± 10.8	31 ± 3.1	Low carbohydrate diet	5	T2DM	CrCl	20% carbohydrate, 30% protein, and 50% fat
		8 (0/8)	63 ± 10.8	31 ± 3.1	Conventional diet				55% carbohydrate, 15% protein, and 30% fat
Rosenvinge Skov et al. 1999 (19)	Denmark	25 (6/19)	39.4 ± 10.0	30.8 ± 2.0	low-protein	26	Uncomplicated obesity	mGFR, UAER	58% carbohydrate, 12% protein, and 30% fat
		25 (6/19)	39.8 ± 9.5	30.8 ± 2.0	High-protein diet				45% carbohydrate, 25% protein, and 30% fat
		15 (3/12)	37.6 ± 8.5	30.3 ± 2.7	Normal-protein diet				NR
Ruggenenti et al. 2016 (30)	Italy	36 (29/7)	60.2 ± 7.2	30 ± 3.9	Calorie restriction	26	T2DM	mGFR, UAER	44.4% carbohydrates, 20.1% protein, 35.7% fat
		38 (27/11)	59.5 ± 7.1	29.6 ± 3.8	Conventional diet				47.5% carbohydrates, 18.3% protein, 34.4% fat
Ruggenenti et al. 2022 (44)	Italy	53 (40/13)	64.9 ± 7.5	32.3 ± 3.7	Calorie restriction	104	T2DM	mGFR, UAER	45–50% carbohydrate, 15–20% protein, and 30–35% fat
		50 (41/9)	62.8 ± 8.7	32.1 ± 3.1	Conventional diet				45–50% carbohydrate, 15–20% protein, and 30–35% fat
Salomo et al. 2016 (45)	Denmark	82 (28/54)	43.9 ± 13.1	30.6 ± 4.8	New Nordic Diet	26	Uncomplicated obesity	CrCl	NR
		50 (16/34)	41.1 ± 13.3	30.4 ± 5.3	Average Danish Diet				NR
Tay et al. 2018 (46)	Australia	58 (37/21)	58 ± 5.6	34.2 ± 3.1	Low carbohydrate diet	104	T2DM	eGFR, UAER	14% carbohydrate, 28% protein, and 58% fat
		57 (29/28)	58 ± 5.2	35.1 ± 2.8	Conventional diet				53% carbohydrate, 17% protein, and 30% fat

NAFLD, non-alcoholic fatty liver disease; MetS, metabolic syndrome; T2DM, type 2 diabetes; eGFR, estimated GFR; mGFR, measured GFR; CrCl, creatinine clearance; NR, not reported.

### 3.2 GFR

**Figure 2:**
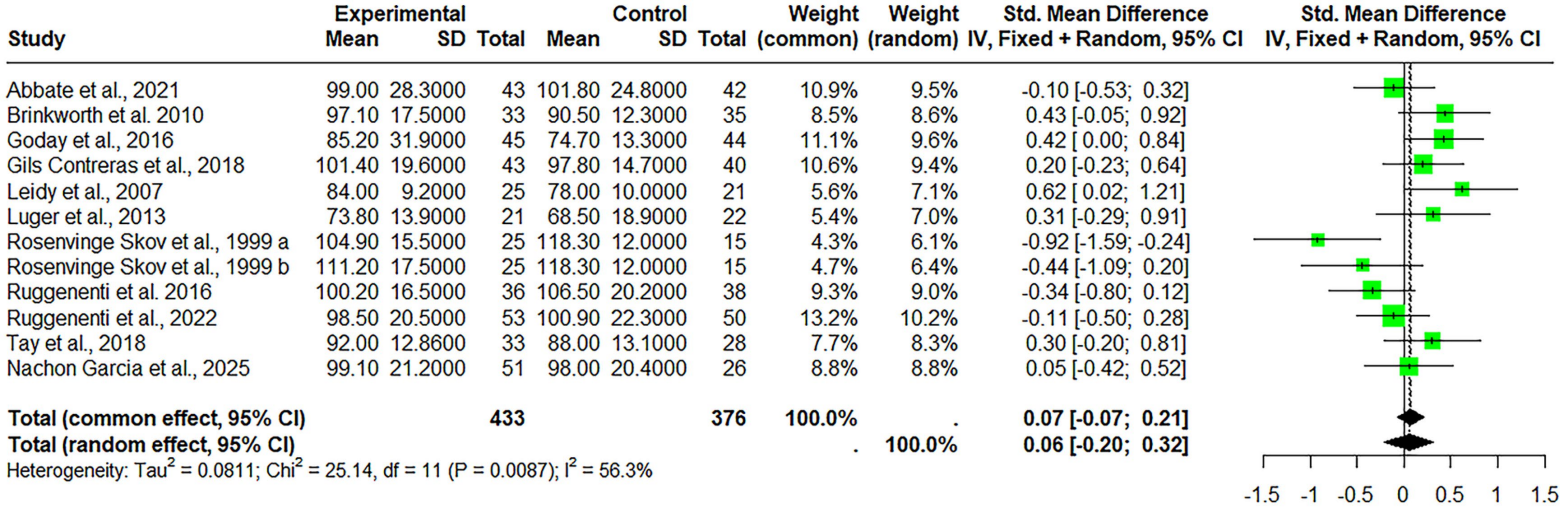
Forest plot of randomized controlled trials evaluating the effect of dietary interventions on glomerular filtration rate (GFR).

**Figure 3:**
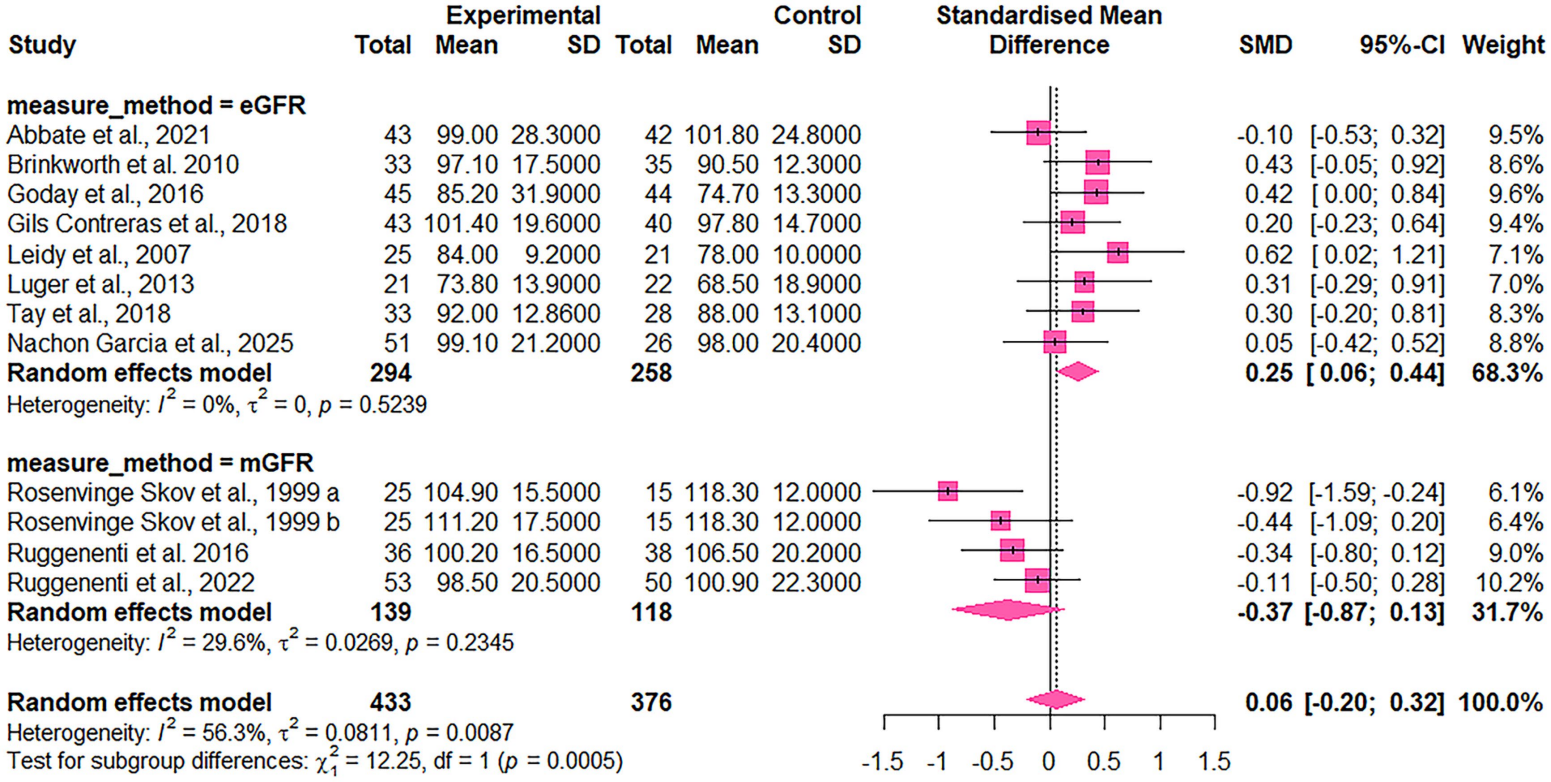
Subgroup analysis of dietary interventions on glomerular filtration rate (GFR) stratified by assessment method (estimated GFR [eGFR] vs. measured GFR [mGFR]).

We analysed GFR from 11 randomized controlled trials, including 809 participants. Dietary interventions showed no significant effect on GFR compared with controls (SMD: 0.06; 95% CI: −0.20 to 0.32; *p* = 0.642; *I*^2^ = 56.3%) (Figure 2). In subgroup analyses stratified by GFR assessment method (eGFR vs. mGFR), dietary interventions were associated with a significant increase in eGFR (SMD: 0.25; 95% CI: 0.06 to 0.44; *p* = 0.017; *I*^2^ = 0%) (Figure 3). In contrast, dietary interventions did not show a significant reduction in mGFR (SMD: −0.37; 95% CI: −0.87 to 0.13; *p* = 0.102; *I*^2^ = 29.6%) (Figure 3). The difference between subgroups was statistically significant (*p* = 0.0005), indicating that the estimated effect of dietary interventions on GFR differed by assessment method and that GFR assessment method may represent a potential source of between-study heterogeneity.

### 3.3 UAER

**Figure 4:**
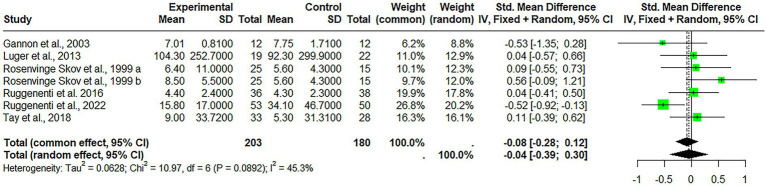
Forest plot of randomized controlled trials evaluating the effect of dietary interventions on urinary albumin excretion rate (UAER).

Six randomized controlled trials reported data on UAER, comprising 383 participants. We found that there was no significant difference between dietary interventions and controls in UAER (SMD: −0.04; 95% CI: −0.39 to 0.30; *p* = 0.765; *I*^2^ = 45.3%) (Figure 4).

### 3.4 CrCl

**Figure 5:**
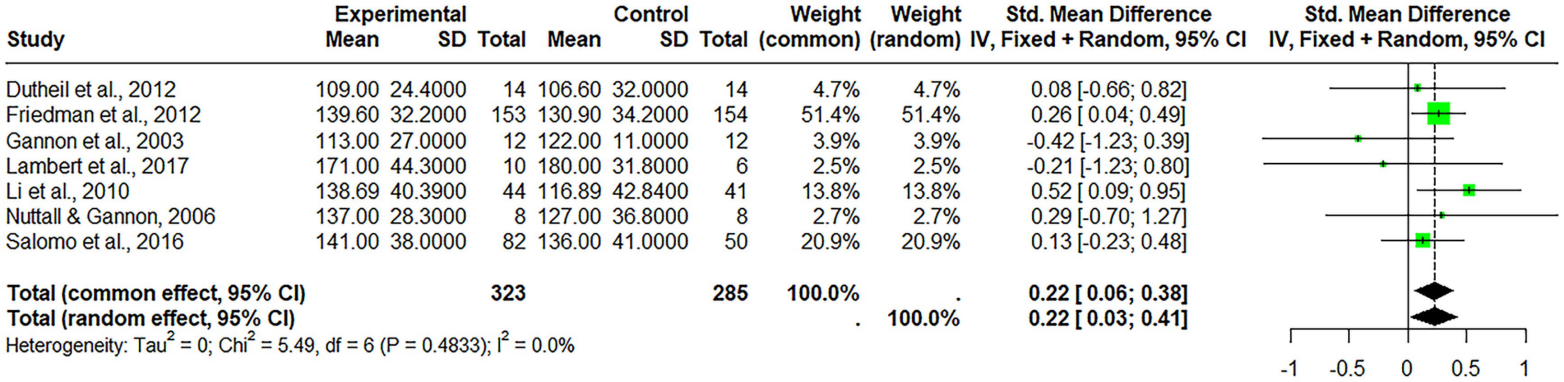
Forest plot of randomized controlled trials evaluating the effect of dietary interventions on creatinine clearance (CrCl).

In total, 7 randomized controlled trials including 608 participants, were included for CrCl. Dietary interventions showed a statistically significant increase in CrCl compared with controls (SMD: 0.22; 95% CI: 0.03 to 0.41; *p* = 0.030; *I*^2^ = 0%) (Figure 5).

### 3.5 Meta-regression

In meta-regression, mean age was not a significant predictor of the intervention effect for GFR (*β* = 0.01 SMD per decade; 95% CI: −0.02 to 0.04; *p* = 0.391) (Supplementary Figure S1). Residual heterogeneity remained after adjustment for age (τ^2^ = 0.095; QE (10) = 24.59, *p* = 0.006), with no heterogeneity explained by the moderator (*R*^2^ analog = 0%). For UAER, studies with higher mean baseline age tended to show greater reductions (*β* = −0.03 SMD per decade; 95% CI: −0.06 to 0.01; *p* = 0.084) (Supplementary Figure S2). Residual heterogeneity was low and non-significant after accounting for age (τ^2^ = 0.017; QE (5) = 5.66, *p* = 0.341), and the model suggested that age explained a substantial proportion of the between-study heterogeneity (*R*^2^ analog = 72.5%). For CrCl, between-study heterogeneity was absent at baseline (τ^2^ = 0, *I*^2^ = 0%), and remained null after inclusion of age (τ^2^ = 0, *I*^2^ = 0%). Consistently, mean age was not associated with the effect of the dietary intervention (*β* = 0.00 SMD per decade; 95% CI: −0.03 to 0.03; *p* = 0.987) (Supplementary Figure S3).

### 3.6 Publication bias and sensitivity analyses

We assessed small study effects/publication bias for GFR (*k* = 12), UAER (*k* = 7), and CrCl (*k* = 7) using contour enhanced funnel plots, Egger’s regression test, and the Duval Tweedie trim-and- fill method. Visual inspection of the funnel plots did not suggest marked asymmetry for GFR or UAER, whereas the CrCl funnel plot showed possible asymmetry. Egger’s tests were not significant for any outcome (GFR: *p* = 0.545; UAER: *p* = 0.392; CrCl: *p* = 0.284). Trim-and-fill imputed no potentially missing studies for GFR (SMD: 0.06; 95% CI: −0.20 to 0.32; *p* = 0.642) (Supplementary Figure S4), one for UAER (SMD: −0.13; 95% CI: −0.50 to 0.24; *p* = 0.438) (Supplementary Figure S5), and two for CrCl (SMD: 0.26; 95% CI: 0.06 to 0.46; *p* = 0.016) (Supplementary Figure S6). After excluding outliers identified in the heterogeneity diagnostics, the pooled estimate and the overall conclusions were materially unchanged.

In addition to heterogeneity diagnostics, we also performed an influence analysis for GFR and UAER, which showed moderate heterogeneity. For GFR, Rosenvinge Skov et al. (19) was identified as an influential study. After excluding this study, the pooled effect remained non-significant (SMD: 0.12; 95% CI: −0.10 to 0.33; *p* = 0.253). For UAER, no high-impact studies were identified.

### 3.7 Quality assessment

**Figure 6:**
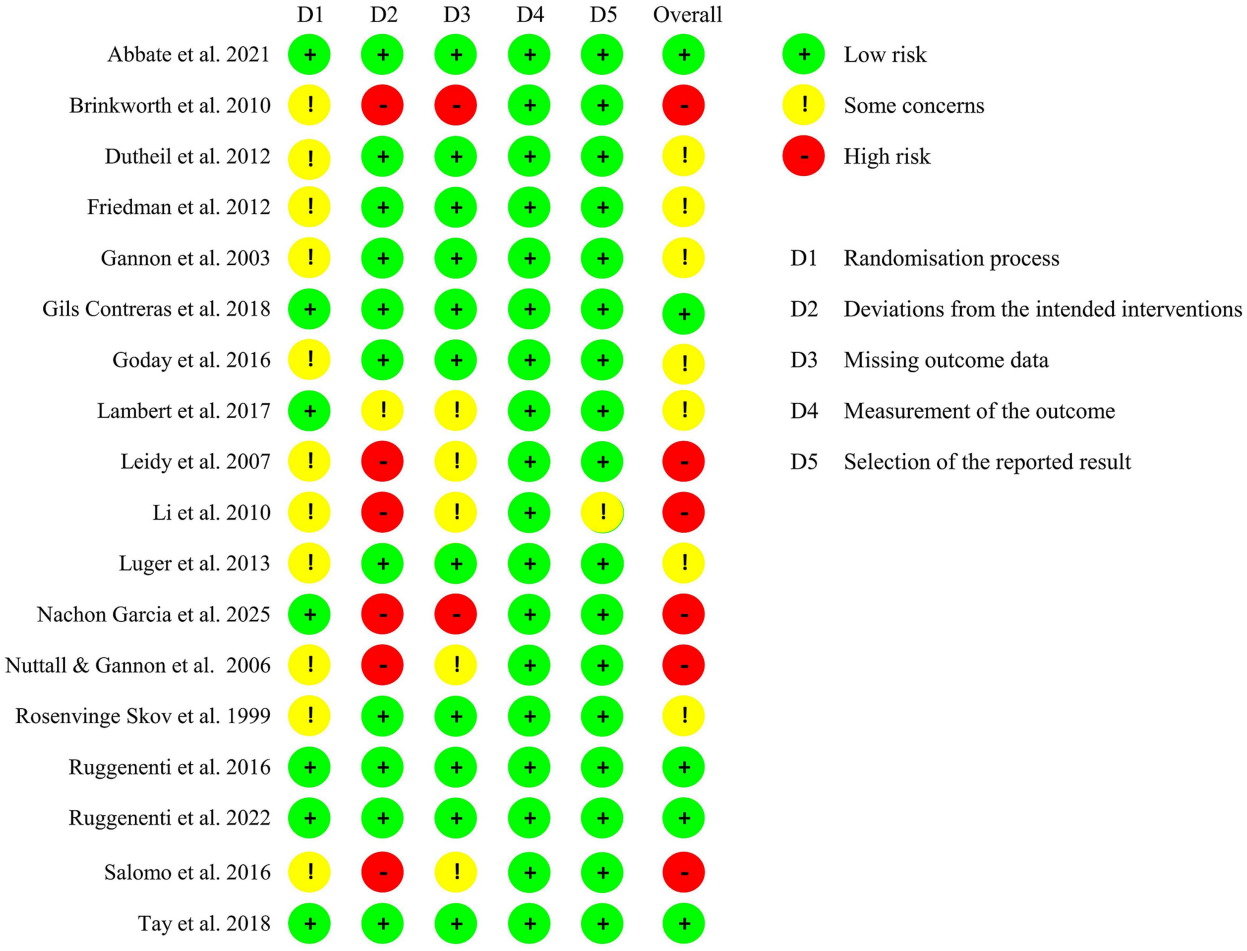
Risk of bias for each included study.

Based on RoB 2 tool, 5 of 18 trials (27.8%) were judged as low risk of bias, 7 trials (38.9%) had some concerns, and 6 trials (33.3%) were judged as high risk of bias (Figure 6). When considering the relative contribution of included studies, trials rated as some concerns accounted for 39.0% of the cumulative weight, while low- and high-risk trials contributed 32.1 and 28.9%, respectively. The risk of bias for each trial is shown in Supplementary Table S3.

The GRADE certainty of evidence ranged from low to very low across outcomes (Supplementary Figure S7). Certainty was low for overall GFR and mGFR, downgraded for risk of bias and imprecision. Certainty was also low for eGFR and CrCl, downgraded for risk of bias and indirectness. Evidence for UAER was rated very low certainty, downgraded for risk of bias, inconsistency, and imprecision.

## 4 Discussion

This meta-analysis evaluated the effects of dietary interventions on kidney function in adults with obesity but without CKD. Our findings showed that dietary interventions were not associated with statistically significant differences in GFR or UAER, but were associated with a small increase in CrCl. Notably, effects on GFR differed by assessment method. eGFR showed a significant increase whereas mGFR showed a non-significant reduction, with a significant between-subgroup difference, suggesting that GFR assessment method influences interpretation and may contribute to between-study heterogeneity.

In contrast to prior meta-analyses of dietary interventions in adults with obesity without CKD, we did not find a significant increase in GFR. This discrepancy is likely attributable to differences in outcome definition: earlier meta-analyses combined CrCl, eGFR, mGFR into a single GFR outcome. Moreover, our subgroup analyses suggested a significant difference between eGFR and mGFR—dietary interventions were associated with a significant increase in eGFR, whereas mGFR showed a non-significant reduction. Notably, CrCl also increased significantly and showed no heterogeneity, aligning with eGFR. Consistent with this pattern, a prospective Roux-en-Y gastric bypass study reported a decline in mGFR alongside increases in creatinine-based eGFR (20). The significant increases in eGFR and CrCl suggest that dietary interventions were associated with favorable changes in kidney function-related indices. However, mGFR, which is less influenced by non-GFR determinants (7), did not show concordant improvement, and the difference between eGFR and mGFR was significant in subgroup analyses. Accordingly, whether the observed increases in eGFR and CrCl translate into improved true glomerular filtration requires further validation. Taken together, the apparent renal effects of dietary intervention depend on how kidney function is assessed.

In the setting of dietary intervention, both creatinine-based and cystatin C-based eGFR may be influenced by non-GFR determinants. Weight loss is associated with reductions in muscle mass, creatinine generation, and serum creatinine concentration, which may lead to an apparent increase in creatinine-based eGFR (11). Similarly, cystatin C–based eGFR may vary with changes in adiposity or BMI (21). For CrCl, because creatinine is filtered by the glomerulus and secreted by the proximal tubule, CrCl systematically overestimates true GFR by approximately 10 to 40% at all levels of GFR (10). In addition, dietary interventions may alter creatinine production, thereby further affecting CrCl independently of true filtration. Therefore, changes in kidney function estimates after dietary intervention should be interpreted in light of these potential non-GFR and measurement-related influences and, where available, alongside mGFR, while also taking into account the biological properties of the filtration marker and the clinical context of the intervention.

**Figure 7:**
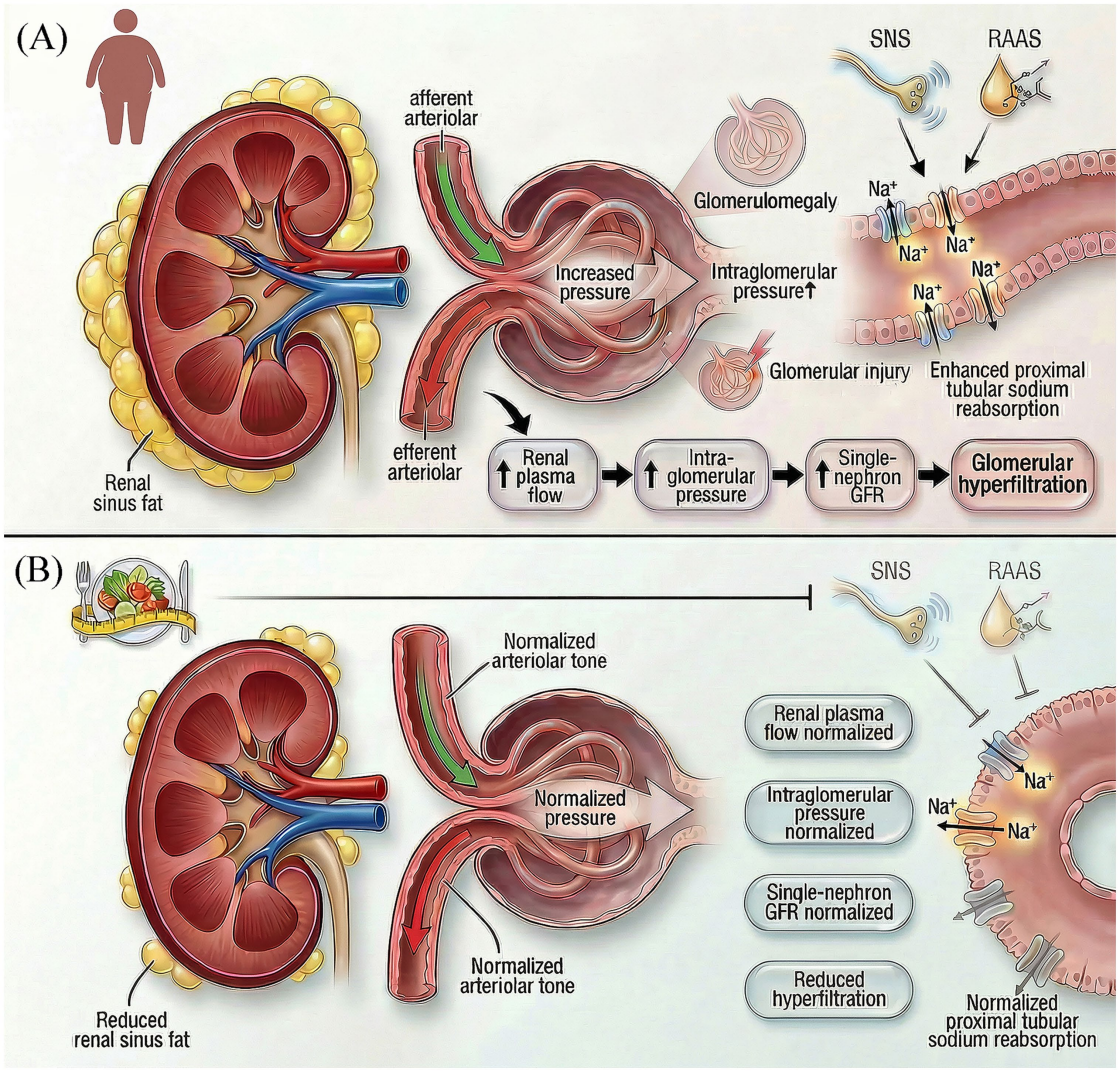
Mechanisms of obesity-related glomerular hyperfiltration and its potential attenuation by dietary intervention. **(A)** Obesity is associated with renal hemodynamic changes and enhanced proximal tubular sodium reabsorption. Activation of neurohormonal pathways such as the renin–angiotensin–aldosterone system (RAAS) and sympathetic nervous system (SNS) contributes to relative efferent arteriolar vasoconstriction. In parallel, increased sodium reabsorption in the proximal tubule weakens tubuloglomerular feedback and promotes afferent arteriolar vasodilation. These combined changes increase renal plasma flow and intraglomerular pressure, thereby raising single-nephron glomerular filtration rate (GFR) and manifesting clinically as glomerular hyperfiltration. Persistent hyperfiltration may lead to glomerulomegaly and increase susceptibility to subsequent glomerular injury. **(B)** Dietary intervention may reduce renal sinus and perirenal fat, thereby alleviating the direct compressive effects of renal adipose tissue on the renal vasculature and parenchyma. In parallel, it may attenuate SNS/RAAS activity and normalize proximal tubular sodium reabsorption. Collectively, these changes may lower renal plasma flow and intraglomerular pressure toward physiologic levels, thereby shifting hyperfiltration toward normofiltration. Created with BioRender.com.

Studies have shown that obesity is associated with a higher prevalence of glomerular hyperfiltration (22, 23). Mechanistically, obesity is associated with renal hemodynamic changes and enhanced proximal tubular sodium reabsorption, which result in afferent arteriolar vasodilation with relative efferent arteriolar vasoconstriction (24, 25). This increases renal plasma flow and raises intraglomerular pressure, thereby increasing single-nephron GFR and manifesting clinically as glomerular hyperfiltration (3, 26). Persistent hyperfiltration is frequently accompanied by glomerulomegaly and may contribute to subsequent glomerular injury (3, 27) (Figure 7A). Importantly, hyperfiltration may represent a potentially reversible physiological state. Dietary interventions might mitigate obesity-related hyperfiltration by reducing renal sinus fat and perirenal fat, thereby alleviating direct compressive effects of renal adipose tissue on renal vasculature and tissue, and normalizing sodium reabsorption in the proximal tubule (28). In parallel, through attenuation of obesity-related sympathetic activity and RAAS activation, dietary interventions may reduce sodium retention and promote efferent arteriolar dilation, jointly lowering intraglomerular pressure and single-nephron workload (24, 29) (Figure 7B).

Consistent with this pathophysiological interpretation, two randomized controlled trials that assessed kidney function using mGFR consistently observed an early decline in mGFR following calorie restriction (19, 30). This decline was more pronounced among participants with baseline hyperfiltration and occurred in parallel with improvements in body weight, waist circumference, blood pressure, and metabolic parameters. Over longer-term follow-up, individuals with obesity who exhibited an early reduction in GFR appeared to have a more stable subsequent GFR trajectory, rather than continued decline or deterioration. Taken together, these findings support the possibility that the potential renal benefit of dietary intervention in some individuals with obesity may not be manifested as an increase in filtration, but rather as attenuation of obesity-related absolute or relative glomerular hyperfiltration. Although the pooled mGFR effect in our analysis did not reach statistical significance, the trend toward lower mGFR may represent normalization from obesity-related hyperfiltration. However, the number of participants contributing to the mGFR analyses was limited, which may have reduced statistical power to detect a significant effect.

Moreover, we did not detect a significant effect of dietary interventions on UAER, suggesting no clear evidence of albuminuria improvement in this population. Because the present study enrolled participants without CKD (AER < 30 mg/24 h or ACR < 30 mg/g), baseline albumin excretion was likely low in most participants, limiting room for detectable improvement. In meta-regression, mean age showed a trend toward greater UAER reductions with increasing age and explained a substantial proportion of between-study heterogeneity. However, the estimated age-related change in effect size was small, and the analysis was based on a limited number of trials. Therefore, this finding should be interpreted cautiously and considered hypothesis-generating.

This meta-analysis has several limitations. First, the number of trials reporting mGFR remains small. This limited sample size restricts statistical power and results in wide confidence intervals, which reduces precision in estimating true changes in filtration and constrains inference on potential normalization of hyperfiltration. Second, the majority of included trials did not stratify participants by baseline glomerular filtration status (hyperfiltration vs. normofiltration), which could dilute pooled estimates. Third, the follow-up durations across most trials were relatively short. This is an important limitation because obesity-related kidney damage may take years to become clinically apparent, particularly in adults without comorbidities or additional established risk factors for kidney disease. Therefore, the available trials may have been too short to detect clinically meaningful changes in kidney function or albuminuria. In addition, the included dietary interventions were heterogeneous in dietary composition, especially protein intake. Because protein intake can affect kidney function indices such as eGFR and CrCl, this heterogeneity may have influenced the pooled estimates and limits the ability to determine whether the observed effects were attributable to dietary intervention overall or to specific dietary patterns.

In adults with obesity without CKD, our findings do not provide consistent evidence that dietary interventions improve measured glomerular filtration or reduce albuminuria. However, dietary interventions were associated with apparent improvements in certain kidney function-related indices (eGFR and CrCl). Importantly, these findings suggest that the apparent renal effects of dietary intervention depend on how kidney function is assessed. In the setting of substantial weight change, increases in eGFR or CrCl should not be directly interpreted as improvements in filtration, as these indices may be affected by non-GFR determinants. At the same time, findings based on mGFR should also be interpreted cautiously, because few trials reported mGFR data and most did not stratify participants by baseline glomerular filtration status. In some individuals with obesity, the renal benefit of dietary intervention may be better understood as attenuation of obesity-related absolute or relative hyperfiltration rather than as an increase in filtration capacity. Therefore, the present findings should be viewed as evidence of measurement-dependent renal effects, rather than as definitive evidence for or against improved kidney function.

However, this inference remains imprecise because relatively few participants contributed mGFR data, and conventional endpoints such as mGFR and albuminuria may be insufficiently sensitive to detect subtle preclinical renal changes over the relatively short follow-up of most included trials. Future studies should prioritize gold-standard mGFR assessment, stratify participants by baseline hyperfiltration status, and evaluate earlier and more sensitive biomarkers of kidney injury or stress to better define the renal effects of dietary intervention and determine whether early attenuation of hyperfiltration translates into long-term renal benefit.

## Data Availability

The original contributions presented in the study are included in the article/Supplementary material, further inquiries can be directed to the corresponding author.
