# Supplementary material for: Effect of dietary interventions on kidney function in adults with obesity without chronic kidney disease: a systematic review and meta-analysis of randomized controlled trials
Source: Front Nutr. 2026 Jun 29;13:1836822. doi: 10.3389/fnut.2026.1836822 (PMC13357197; doi:10.3389/fnut.2026.1836822)
Supplement: Supplementary file 1 [file Table_1.DOCX]

Supplementary Material

**Supplementary Table 1.** PRISMA 2020 Checklist

| **Section and Topic** | **Item #** | **Checklist item** | **Location where item is reported** |
| --- | --- | --- | --- |
| **TITLE** | | |  |
| Title | 1 | Identify the report as a systematic review. |  |
| **ABSTRACT** | | |  |
| Abstract | 2 | See the PRISMA 2020 for Abstracts checklist. |  |
| **INTRODUCTION** | | |  |
| Rationale | 3 | Describe the rationale for the review in the context of existing knowledge. |  |
| Objectives | 4 | Provide an explicit statement of the objective(s) or question(s) the review addresses. |  |
| **METHODS** | | |  |
| Eligibility criteria | 5 | Specify the inclusion and exclusion criteria for the review and how studies were grouped for the syntheses. |  |
| Information sources | 6 | Specify all databases, registers, websites, organisations, reference lists and other sources searched or consulted to identify studies. Specify the date when each source was last searched or consulted. |  |
| Search strategy | 7 | Present the full search strategies for all databases, registers and websites, including any filters and limits used. |  |
| Selection process | 8 | Specify the methods used to decide whether a study met the inclusion criteria of the review, including how many reviewers screened each record and each report retrieved, whether they worked independently, and if applicable, details of automation tools used in the process. |  |
| Data collection process | 9 | Specify the methods used to collect data from reports, including how many reviewers collected data from each report, whether they worked independently, any processes for obtaining or confirming data from study investigators, and if applicable, details of automation tools used in the process. |  |
| Data items | 10a | List and define all outcomes for which data were sought. Specify whether all results that were compatible with each outcome domain in each study were sought (e.g. for all measures, time points, analyses), and if not, the methods used to decide which results to collect. |  |
|  | 10b | List and define all other variables for which data were sought (e.g. participant and intervention characteristics, funding sources). Describe any assumptions made about any missing or unclear information. |  |
| Study risk of bias assessment | 11 | Specify the methods used to assess risk of bias in the included studies, including details of the tool(s) used, how many reviewers assessed each study and whether they worked independently, and if applicable, details of automation tools used in the process. |  |
| Effect measures | 12 | Specify for each outcome the effect measure(s) (e.g. risk ratio, mean difference) used in the synthesis or presentation of results. |  |
| Synthesis methods | 13a | Describe the processes used to decide which studies were eligible for each synthesis (e.g. tabulating the study intervention characteristics and comparing against the planned groups for each synthesis (item #5)). |  |
|  | 13b | Describe any methods required to prepare the data for presentation or synthesis, such as handling of missing summary statistics, or data conversions. |  |
|  | 13c | Describe any methods used to tabulate or visually display results of individual studies and syntheses. |  |
|  | 13d | Describe any methods used to synthesize results and provide a rationale for the choice(s). If meta-analysis was performed, describe the model(s), method(s) to identify the presence and extent of statistical heterogeneity, and software package(s) used. |  |
|  | 13e | Describe any methods used to explore possible causes of heterogeneity among study results (e.g. subgroup analysis, meta-regression). |  |
|  | 13f | Describe any sensitivity analyses conducted to assess robustness of the synthesized results. |  |
| Reporting bias assessment | 14 | Describe any methods used to assess risk of bias due to missing results in a synthesis (arising from reporting biases). |  |
| Certainty assessment | 15 | Describe any methods used to assess certainty (or confidence) in the body of evidence for an outcome. |  |
| **RESULTS** | | |  |
| Study selection | 16a | Describe the results of the search and selection process, from the number of records identified in the search to the number of studies included in the review, ideally using a flow diagram. |  |
|  | 16b | Cite studies that might appear to meet the inclusion criteria, but which were excluded, and explain why they were excluded. |  |
| Study characteristics | 17 | Cite each included study and present its characteristics. |  |
| Risk of bias in studies | 18 | Present assessments of risk of bias for each included study. |  |
| Results of individual studies | 19 | For all outcomes, present, for each study: (a) summary statistics for each group (where appropriate) and (b) an effect estimate and its precision (e.g. confidence/credible interval), ideally using structured tables or plots. |  |
| Results of syntheses | 20a | For each synthesis, briefly summarise the characteristics and risk of bias among contributing studies. |  |
|  | 20b | Present results of all statistical syntheses conducted. If meta-analysis was done, present for each the summary estimate and its precision (e.g. confidence/credible interval) and measures of statistical heterogeneity. If comparing groups, describe the direction of the effect. |  |
|  | 20c | Present results of all investigations of possible causes of heterogeneity among study results. |  |
|  | 20d | Present results of all sensitivity analyses conducted to assess the robustness of the synthesized results. |  |
| Reporting biases | 21 | Present assessments of risk of bias due to missing results (arising from reporting biases) for each synthesis assessed. |  |
| Certainty of evidence | 22 | Present assessments of certainty (or confidence) in the body of evidence for each outcome assessed. |  |
| **DISCUSSION** | | |  |
| Discussion | 23a | Provide a general interpretation of the results in the context of other evidence. |  |
|  | 23b | Discuss any limitations of the evidence included in the review. |  |
|  | 23c | Discuss any limitations of the review processes used. |  |
|  | 23d | Discuss implications of the results for practice, policy, and future research. |  |
| **OTHER INFORMATION** | | |  |
| Registration and protocol | 24a | Provide registration information for the review, including register name and registration number, or state that the review was not registered. |  |
|  | 24b | Indicate where the review protocol can be accessed, or state that a protocol was not prepared. |  |
|  | 24c | Describe and explain any amendments to information provided at registration or in the protocol. |  |
| Support | 25 | Describe sources of financial or non-financial support for the review, and the role of the funders or sponsors in the review. |  |
| Competing interests | 26 | Declare any competing interests of review authors. |  |
| Availability of data, code and other materials | 27 | Report which of the following are publicly available and where they can be found: template data collection forms; data extracted from included studies; data used for all analyses; analytic code; any other materials used in the review. |  |

**Supplementary Table 2.** Search strategy

| **#** | **Embase** |
| --- | --- |
| 1 | obesity'/exp |
| 2 | adipose tissue hyperplasia':ab,ti OR 'adipositas':ab,ti OR 'adiposity':ab,ti OR 'alimentary obesity':ab,ti OR 'body weight, excess':ab,ti OR 'corpulency':ab,ti OR 'fat overload syndrome':ab,ti OR 'nutritional obesity':ab,ti OR 'obesitas':ab,ti OR 'overweight':ab,ti OR 'obesity':ab,ti |
| 3 | #1 OR #2 |
| 4 | diet'/exp |
| 5 | diet influence':ab,ti OR 'diet regimen':ab,ti OR 'diet surveys':ab,ti OR 'dietary effect':ab,ti OR 'dietary influence':ab,ti OR 'dietary regimen':ab,ti OR 'dietary survey':ab,ti OR 'dietary surveys':ab,ti OR 'dieting' OR 'diet':ab,ti |
| 6 | #4 OR #5 |
| 7 | kidney function'/exp |
| 8 | function, kidney':ab,ti OR 'kidney physiology':ab,ti OR 'renal function':ab,ti OR 'kidney function':ab,ti OR 'glomerulus filtration rate':ab,ti OR 'albuminuria':ab,ti OR 'urinary albumin excretion rate':ab,ti OR 'urinary albumin excretion':ab,ti OR 'albumin excretion rate':ab,ti OR 'creatinine':ab,ti OR 'creatinine clearance':ab,ti |
| 9 | #7 OR #8 |
| 10 | randomized controlled trial'/exp |
| 11 | randomization':ab,ti OR 'double blind procedure':ab,ti OR 'rct':ab,ti |
| 12 | #10 OR #11 |
| 13 | #3 AND #6 AND #9 AND #12 |

| **#** | **cochrane library** |
| --- | --- |
| 1 | MeSH descriptor: [Diet] explode all trees |
| 2 | (Diets):ti,ab,kw OR (Dietary Pattern):ti,ab,kw OR (Diet Therapy):ti,ab,kw |
| 3 | #1 OR #2 |
| 4 | MeSH descriptor: [Obesity] explode all trees |
| 5 | (Obesity):ti,ab,kw OR (Obese):ti,ab,kw OR (Obesity, Abdominal):ti,ab,kw OR (Obesity, metabolically benign):ti,ab,kw OR (Obesity, Morbid):ti,ab,kw OR (Overweight):ti,ab,kw OR (Overweight):ti,ab,kw OR (Over weight):ti,ab,kw |
| 6 | #4 OR #5 |
| 7 | MeSH descriptor: [Kidney Function Tests] explode all trees |
| 8 | (Kidney Function):ti,ab,kw OR (Renal Function):ti,ab,kw OR (Glomerular Filtration Rate):ti,ab,kw OR (Albuminuria):ti,ab,kw OR (Urinary albumin excretion rate):ti,ab,kw OR (Urinary albumin excretion):ti,ab,kw OR (Albumin excretion rate):ti,ab,kw OR (Creatinine):ti,ab,kw OR (Creatinine clearance):ti,ab,kw |
| 9 | #7 OR #8 |
| 10 | MeSH descriptor: [Randomized Controlled Trial] explode all trees |
| 11 | (Random Allocation):ti,ab,kw OR (Double-Blind Method):ti,ab,kw OR (RCT):ti,ab,kw OR (Randomly):ti,ab,kw OR (Randomization):ti,ab,kw |
| 12 | #10 OR #11 |
| 13 | #3 AND #6 AND #9 AND #12 |

| **#** | **PubMed** |
| --- | --- |
| 1 | (Obesity[MeSH Terms]) |
| 2 | (Obese[Title/Abstract]) OR (Obesity, Abdominal[Title/Abstract]) OR (Obesity, metabolically benign[Title/Abstract]) OR (Obesity, Morbid[Title/Abstract]) OR (Overweight[MeSH Terms]) OR (overweight[Title/Abstract]) OR (over weight[Title/Abstract]) |
| 3 | #1 OR #2 |
| 4 | (Diet[MeSH Terms]) |
| 5 | (Diets[Title/Abstract]) OR (Dietary Pattern[Title/Abstract]) OR (Diet Therapy[Title/Abstract]) |
| 6 | #4 OR #5 |
| 7 | (Kidney Function Tests[MeSH Terms]) |
| 8 | (Kidney Function[Title/Abstract]) OR (Renal Function[Title/Abstract]) OR (Glomerular Filtration Rate[Title/Abstract]) OR (Albuminuria[Title/Abstract]) OR (Urinary albumin excretion rate[Title/Abstract]) OR (Urinary albumin excretion[Title/Abstract]) OR (Albumin excretion rate[Title/Abstract]) OR (Creatinine[Title/Abstract]) OR (Creatinine clearance [Title/Abstract]) |
| 9 | #7 OR #8 |
| 10 | (Randomized Controlled Trial[Publication Type]) |
| 11 | (Random Allocation) OR (Double-Blind Method) OR (RCT) OR (Randomly) OR (Randomization) |
| 12 | #10 OR #11 |
| 13 | #3 AND #6 AND #9 AND #12 |

| **#** | **Web of Science** |
| --- | --- |
| 1 | TS=(Obesity OR obese OR Obesity, Abdominal OR Obesity, metabolically benign OR Obesity, Morbid OR overweight OR over weight) |
| 2 | TS=(Diet OR Diets OR Dietary Pattern OR Diet Therapy) |
| 3 | TS=(Kidney Function OR Renal Function OR Glomerular Filtration Rate OR Albuminuria OR Urinary albumin excretion rate OR Urinary albumin excretion OR Albumin excretion rate OR Creatinine OR Creatinine clearance) |
| 4 | TS=(Randomized Controlled Trial OR Random Allocation OR Double-Blind Method OR RCT OR Randomly OR Randomization) |
| 5 | #1 AND #2 AND #3 AND #4 |

**Supplementary Table 3.** Risk of bias for each trial.

| **Study** | **D1** | **D2** | **D3** | **D4** | **D5** | **overall** |
| --- | --- | --- | --- | --- | --- | --- |
| Abbate et al. 2021 | Low | Low | Low | Low | Low | Low |
| Brinkworth et al. 2010 | Some concerns | High | High | Low | Low | High |
| Dutheil et al. 2012 | Some concerns | Low | Low | Low | Low | Some concerns |
| Friedman et al. 2012 | Some concerns | Low | Low | Low | Low | Some concerns |
| Gannon et al. 2003 | Some concerns | Low | Low | Low | Low | Some concerns |
| Gils Contreras et al. 2018 | Low | Low | Low | Low | Low | Low |
| Goday et al. 2016 | Some concerns | Low | Low | Low | Low | Some concerns |
| Lambert et al. 2017 | Low | Some concerns | Some concerns | Low | Low | Some concerns |
| Leidy et al. 2007 | Some concerns | High | Some concerns | Low | Low | High |
| Li et al. 2010 | Some concerns | High | Some concerns | Low | Some concerns | High |
| Luger et al. 2013 | Some concerns | Low | Low | Low | Low | Some concerns |
| Nachon Garcia et al. 2025 | Low | High | High | Low | Low | High |
| Nuttall & Gannon et al. 2006 | Some concerns | High | Some concerns | Low | Low | High |
| Rosenvinge Skov et al. 1999 | Some concerns | Low | Low | Low | Low | Some concerns |
| Ruggenenti et al. 2016 | Low | Low | Low | Low | Low | Low |
| Ruggenenti et al. 2022 | Low | Low | Low | Low | Low | Low |
| Salomo et al. 2016 | Some concerns | High | Some concerns | Low | Low | High |
| Tay et al. 2018 | Low | Low | Low | Low | Low | Low |


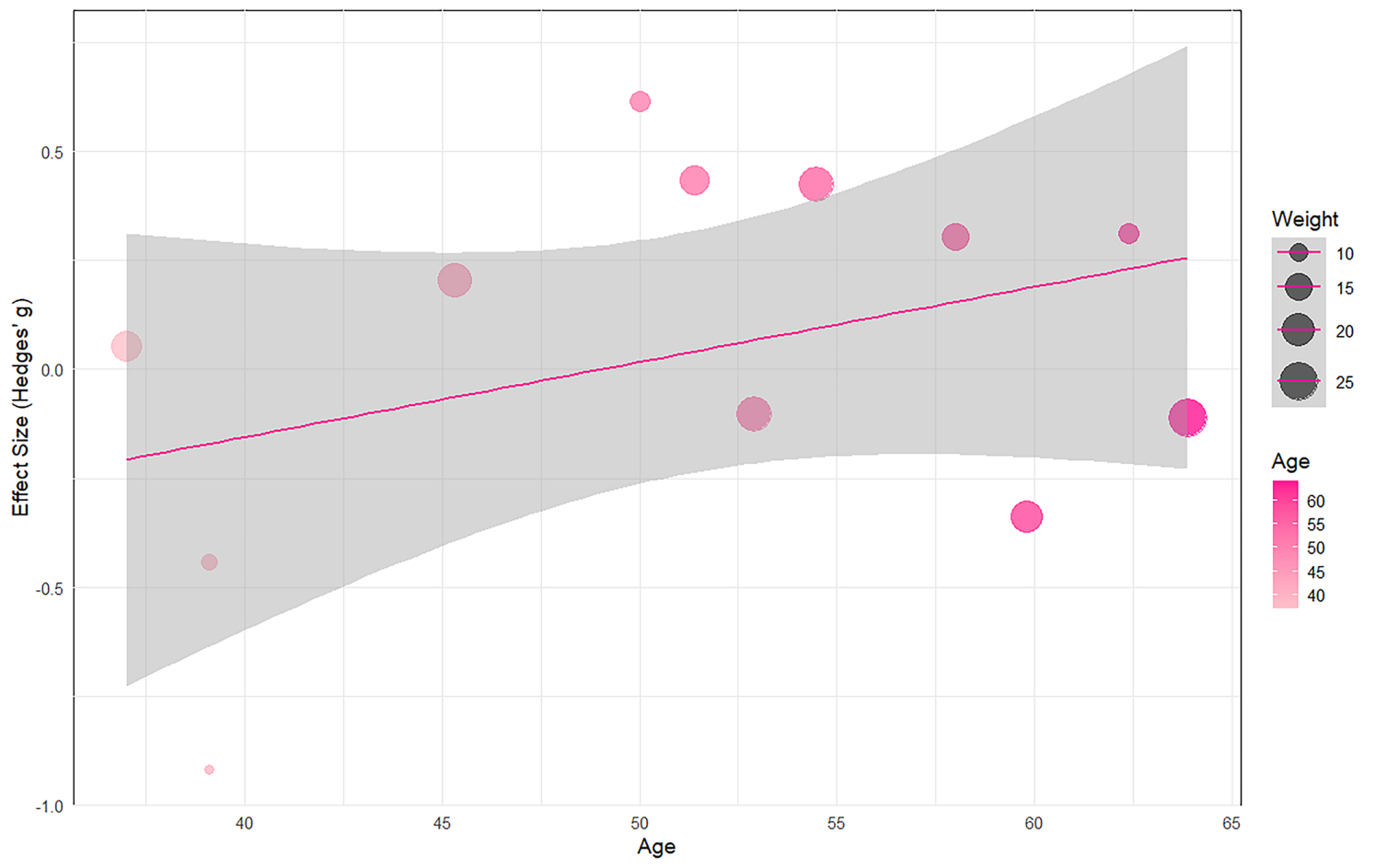


**Supplementary Figure 1.** Meta-regression of intervention effects on GFR by mean baseline age (per 10-year increase). This plot shows the relationship between age and the standardized mean difference (SMD) of the intervention effect, with bubble size representing study precision (1/SE). The pink line indicates the fitted random-effects meta-regression, and the shaded area shows the 95% CI.


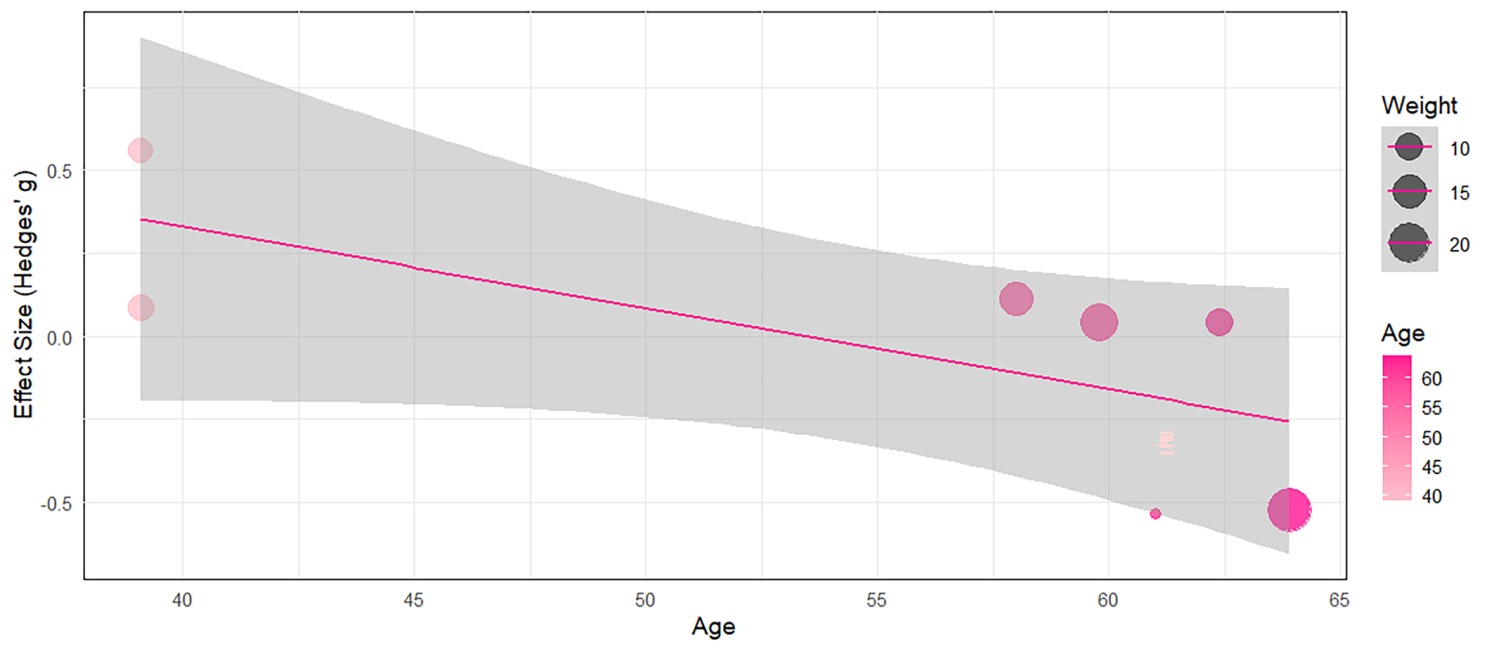


**Supplementary Figure 2.** Meta-regression of intervention effects on UAER by mean baseline age (per 10-year increase). This plot shows the relationship between age and the standardized mean difference (SMD) of the intervention effect, with bubble size representing study precision (1/SE). The pink line indicates the fitted random-effects meta-regression, and the shaded area shows the 95% CI.


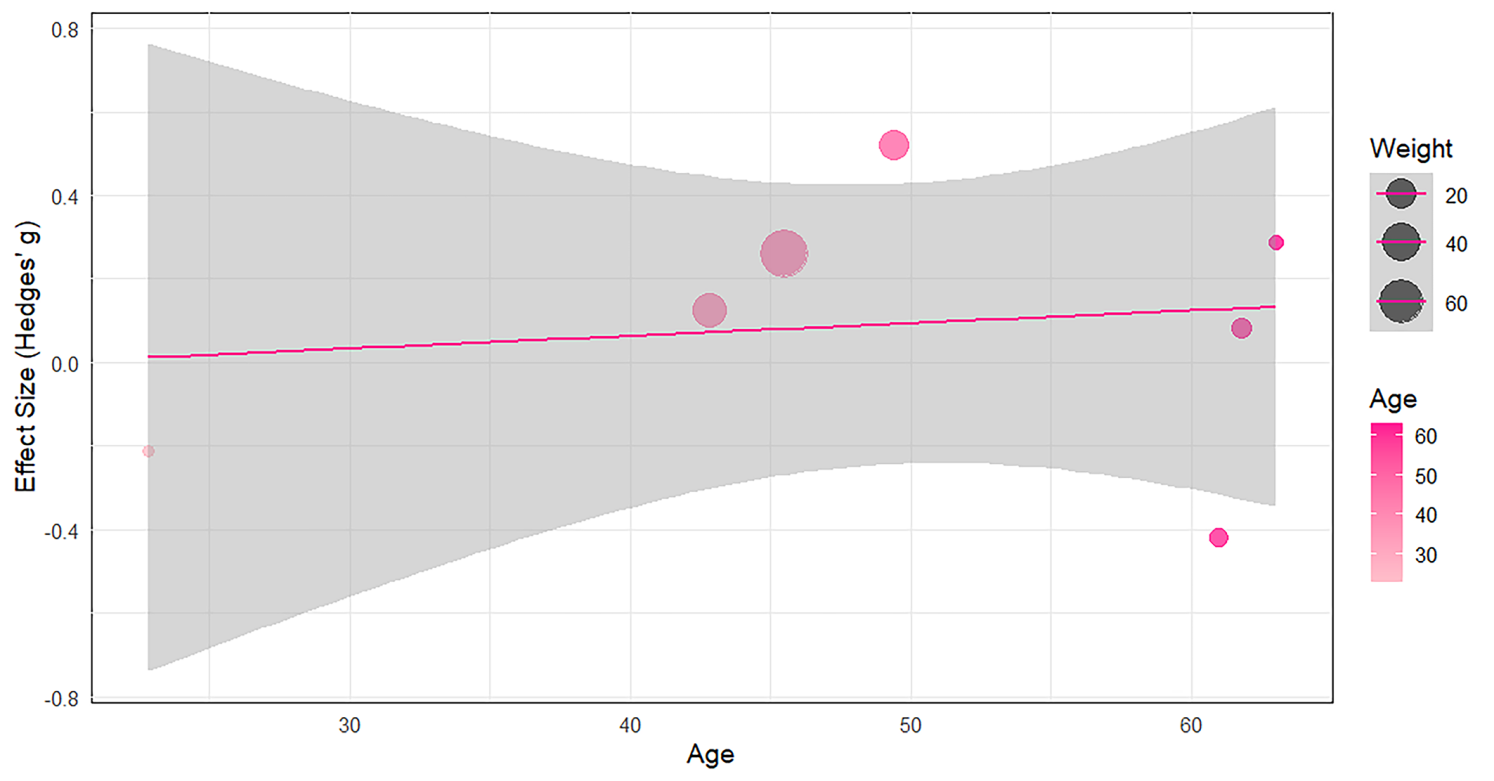


**Supplementary Figure 3.** Meta-regression of intervention effects on CrCl by mean baseline age (per 10-year increase). This plot shows the relationship between age and the standardized mean difference (SMD) of the intervention effect, with bubble size representing study precision (1/SE). The pink line indicates the fitted random-effects meta-regression, and the shaded area shows the 95% CI.


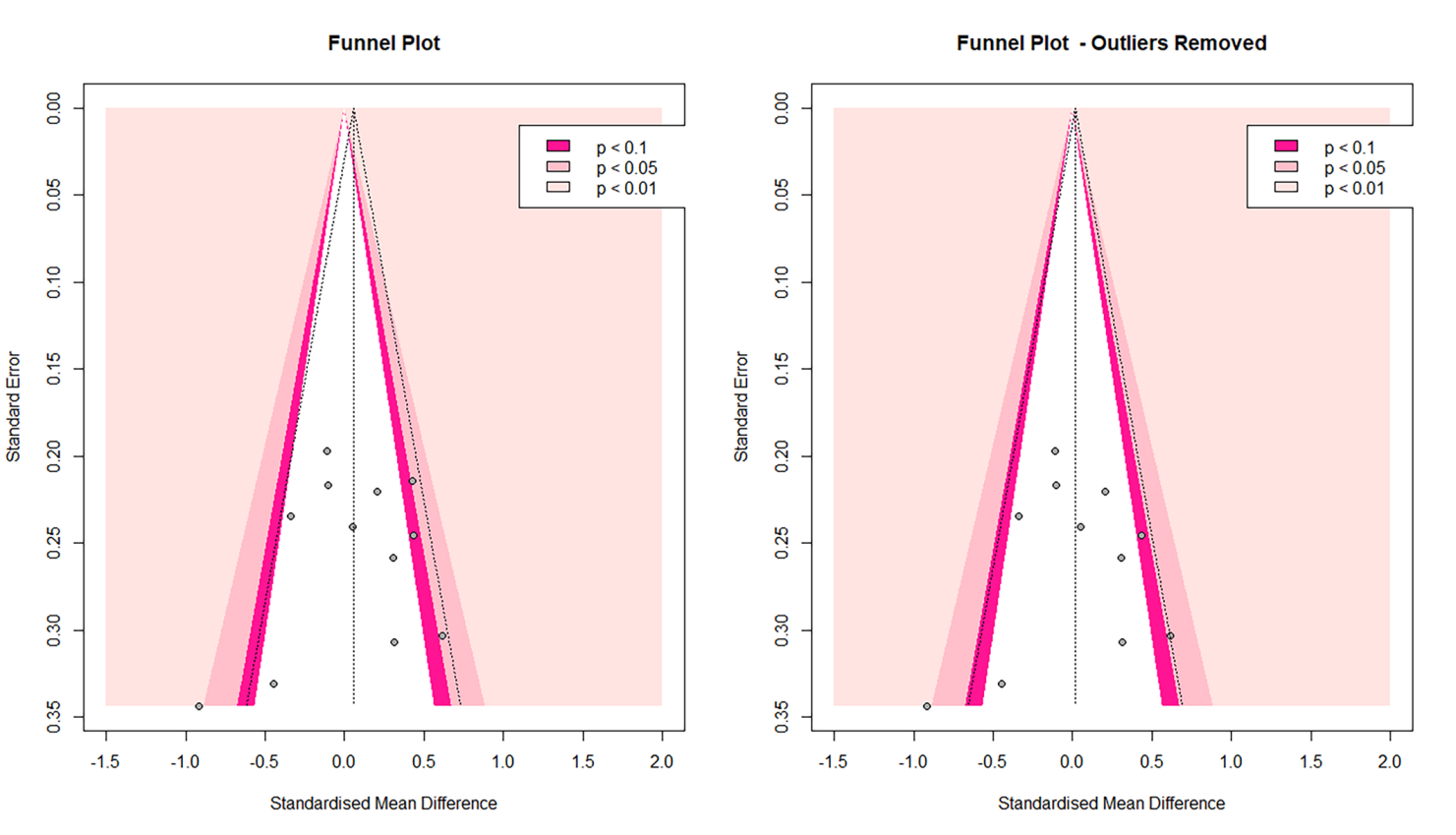


**Supplementary Figure 4.** Contour-enhanced funnel plots assessing small-study effects for dietary interventions versus usual diet on GFR. Left panels show all included studies, and right panels show the funnel plots after removal of outlier or highly influential studies identified by influence diagnostics. The x-axis indicates the standardized mean difference (SMD) and the y-axis the standard error.


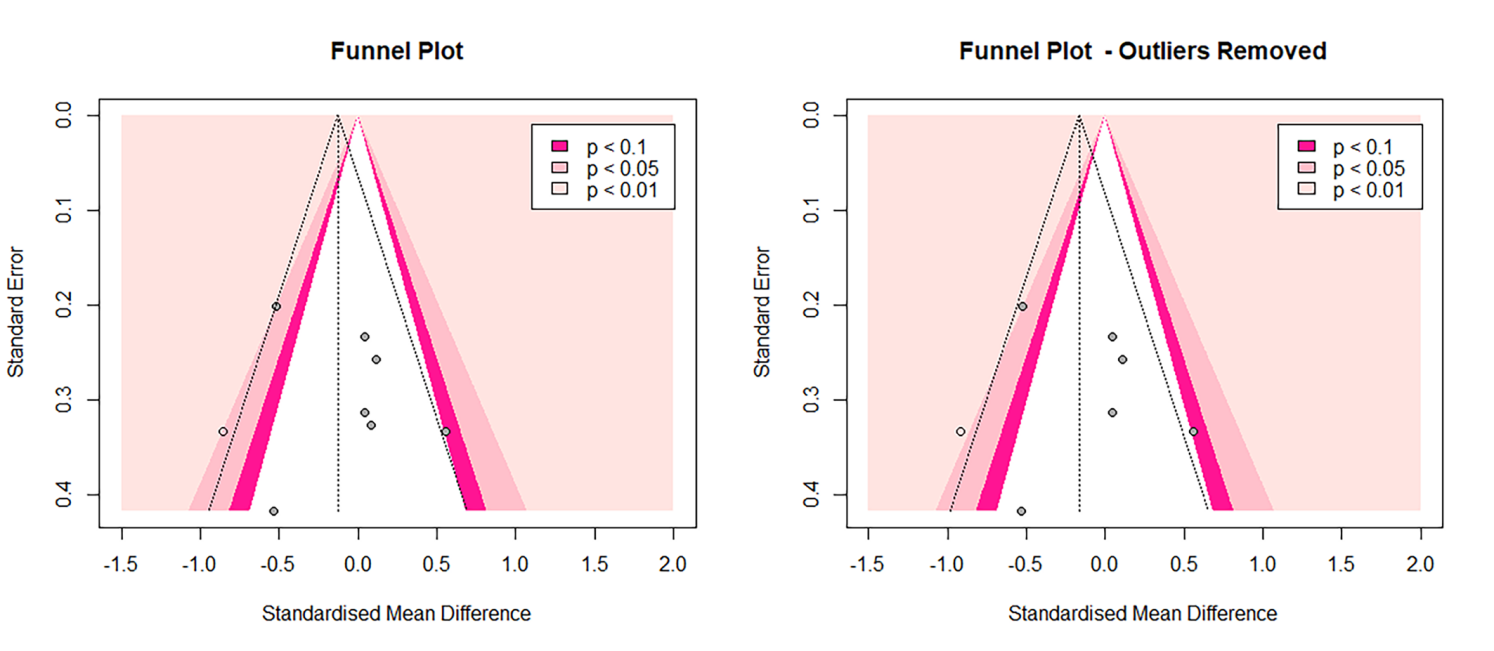


**Supplementary Figure 5.** Contour-enhanced funnel plots assessing small-study effects for dietary interventions versus usual diet on UAER. Left panels show all included studies, and right panels show the funnel plots after removal of outlier or highly influential studies identified by influence diagnostics. The x-axis indicates the standardized mean difference (SMD) and the y-axis the standard error.


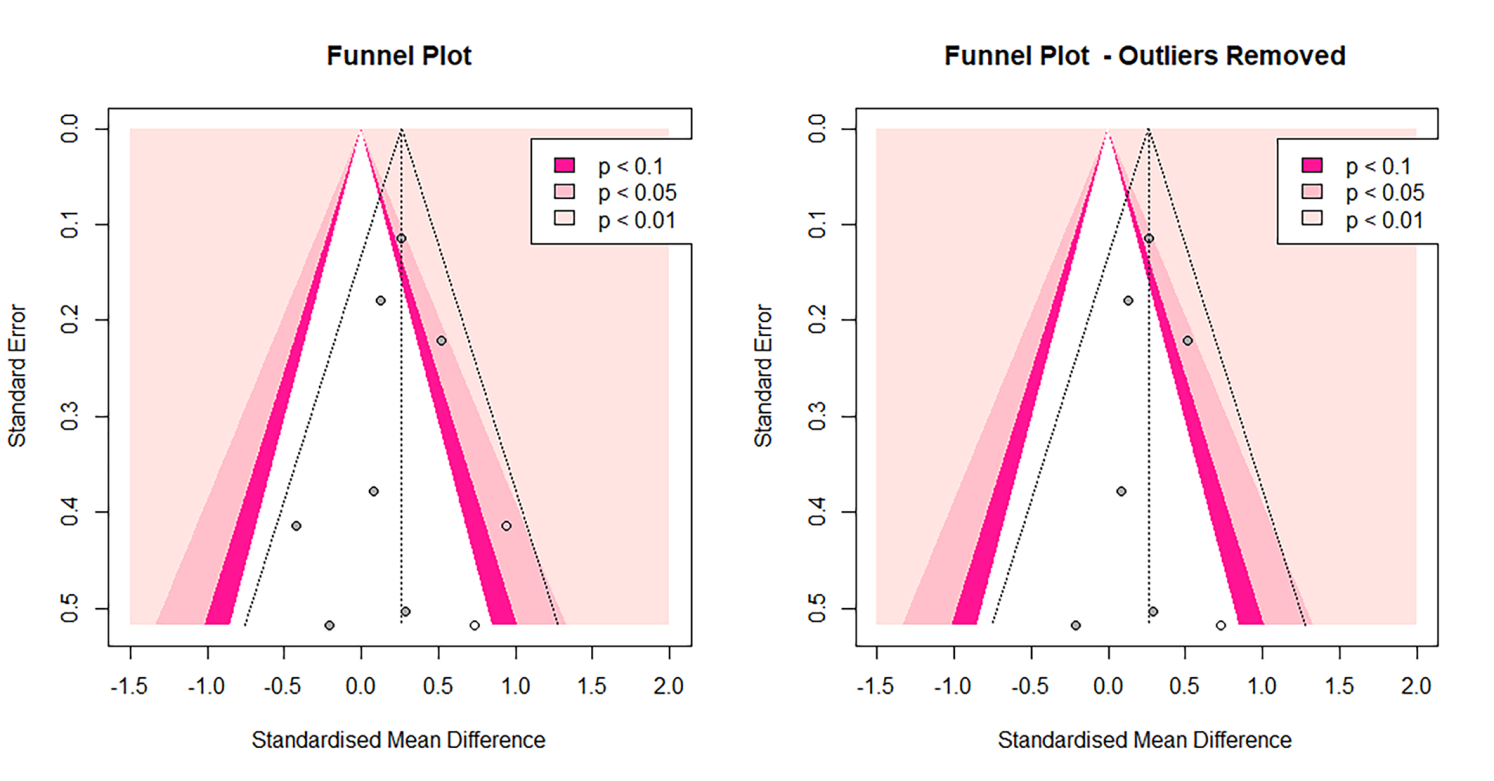


**Supplementary Figure 6.** Contour-enhanced funnel plots assessing small-study effects for dietary interventions versus usual diet on CrCl. Left panels show all included studies, and right panels show the funnel plots after removal of outlier or highly influential studies identified by influence diagnostics. The x-axis indicates the standardized mean difference (SMD) and the y-axis the standard error.


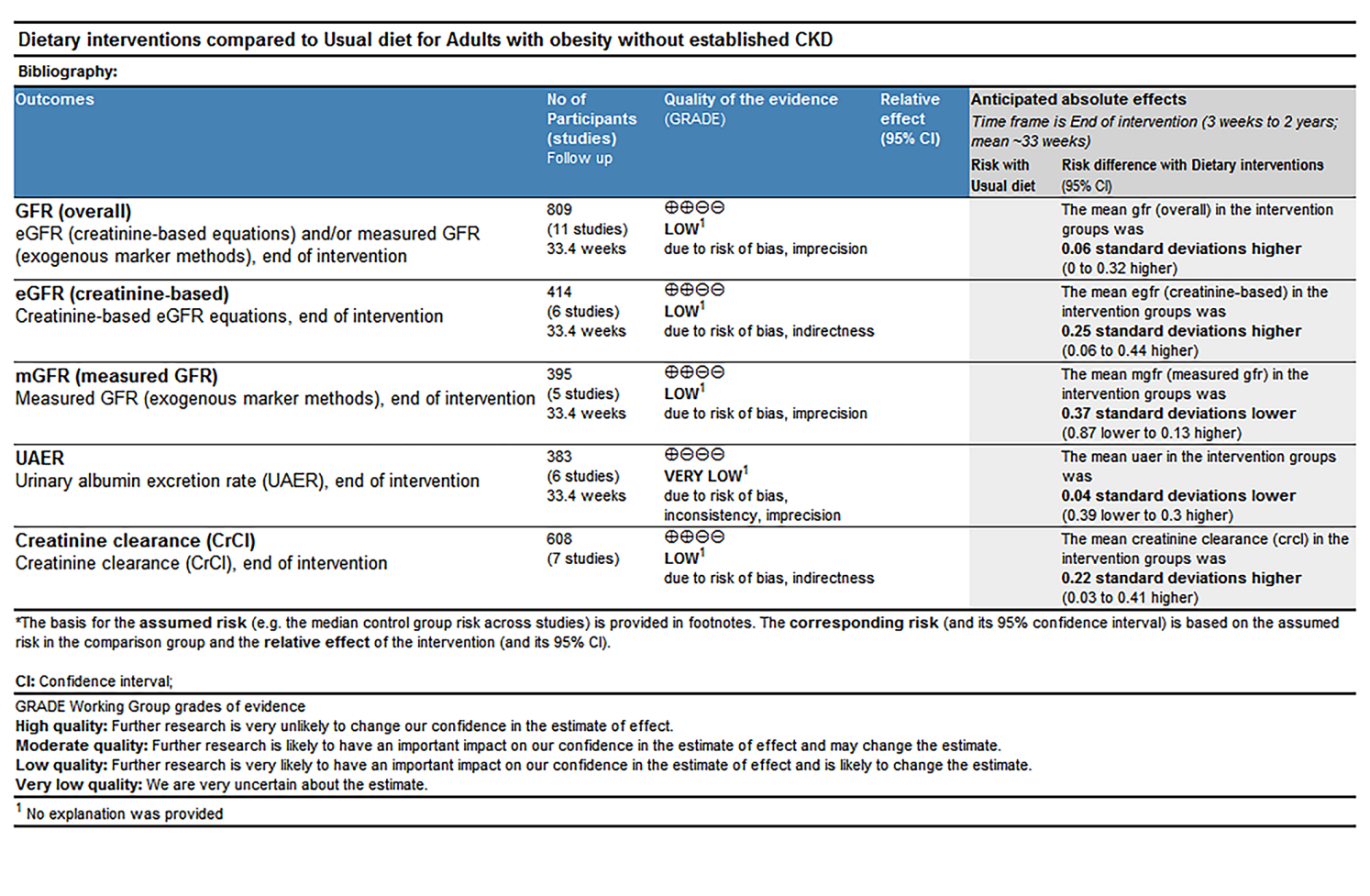


**Supplementary Figure 7.** GRADE assessment of evidence quality.
